# The effect of ascending- descending ultrafiltration and sodium profiles on blood pressure in hemodialysis patients: a randomized cross-over study

**DOI:** 10.1186/s12882-024-03554-6

**Published:** 2024-04-11

**Authors:** Morteza Arasnezhad, Mohammad Namazinia, Seyyed Reza Mazlum, Kheizaran Miri

**Affiliations:** 1 9 Dey Educational Hospital, Torbat Heydariyeh University of Medical Sciences, Torbat Heydariyeh, Iran; 2 Department of Nursing, School of Nursing and Midwifery, Torbat Heydariyeh University of Medical Sciences, Torbat Heydariyeh, Iran; 3 Department of Medical Surgical Nursing, School of Nursing and Midwifery, Mashhad University of Medical Sciences, Mashhad, Iran

**Keywords:** Hemodialysis, Ascending/descending ultrafiltration profile, Sodium profile, Blood pressure

## Abstract

**Background:**

Considering no previous research into the utilization of ascending/descending ultrafiltration and linear sodium profiles in improving blood pressure among hemodialysis patients, the present study aimed to explore the effect of the A/D-UF along with linear sodium profiles on HD patients with hypotension.

**Methods:**

Applying a crossover design, this clinical trial was fulfilled between December 2022 and June 2023 on 20 patients undergoing HD, randomized into two groups, each one receiving two intervention protocols, viz., (a) an intervention protocol in which the liquid sodium in the dialysis solution was linear and the UF profiling was A/D, and (b) a routine protocol or HD, wherein both liquid sodium and UF in the dialysis solution remained constant. The HD patients’ BP was then checked and recorded at six intervals, namely, before HD, one, two, three, and four hours after it, and following its completion, within each session. The data were further statistically analyzed using the IBM SPSS Statistics 20 and the related tests.

**Results:**

In total, 20 patients, including 12 men (60%) and 8 women (40%), with the mean age of 58.00 ± 14.54 on HD for an average of 54 months, were recruited in this study. No statistically significant difference was observed in the mean systolic and diastolic BP levels in the group receiving the A/D-UF profile all through the desired hours (*p* > 0.05), indicating that the patients did not face many changes in these two numbers during HD. Our cross-over clinical trial demonstrated a statistically significant reduction in symptomatic IDH episodes from 55 to 15% with the application of the A/D-UF profile (*p* < 0.05).

**Conclusion:**

The study demonstrated that the A/D-UF profile could contribute to the stability of blood pressure levels among HD patients, with no significant fluctuations observed during treatment sessions.

**Trial Registration:**

This study was registered in the Iranian Registry of Clinical Trials (no. IRCT20180429039463N5) on 07/01/2023.

## Introduction

Chronic kidney disease (CKD) is a growing long-term condition affecting around 2–3% of the global population [1]. The number of hemodialysis (HD) patients in Iran has shown a significant increase over the years, from 945 patients in 1997 to 30,882 cases in 2017 [2]. HD is the most common treatment for CKD and relies on the principles of diffusion and ultrafiltration (UF) [3]. Dialyzer machines help filter waste products, excess fluids, and toxins from the blood of individuals with kidney dysfunction [4]. During UF, fluids are typically removed from the extracellular space (ECF), allowing patients to achieve their dry weight and reduce total plasma volume [5]. However, this treatment can lead to hemodynamic instability, particularly in patients with fluid overload and hypotension prior to HD. These patients may experience hypotension and various symptoms such as headache, dizziness, nausea, vomiting, and decreased consciousness during the later stages of HD [6].

The reported incidence of Intradialytic hypotension ranges from 7.5 to 69% according to different definitions [7, 8]. Of note, hypotension dramatically increases the total number of deaths in such patients, limits fluid withdrawal during HD, and even brings about severe vascular effects, such as a cerebral infarction, as well as cardiac or mesenteric ischemia. Furthermore, it calls for more nursing care services, and has various negative effects on the quality of life of HD patients [9]. The routine intervention protocols practiced at some stage in hypotension during HD correspondingly take account of making some changes in the patient’s position into the Trendelenburg one, moderating or halting the UF process, administering normal saline to restore intravascular volume, using high sodium concentrations, and lowering the dialysate temperature [10–12]. In spite of this, these interventions can result in more sodium and fluid retention in the patient’s body within certain circumstances, wherein they fail to reach dry weight [13]. Among the methods mainly exploited to improve blood pressure (BP) is UF profiling [14], described as a set of programs to change the UF speed at different time intervals based on patient’s condition, characterized by assorted types, i.e., linear, step-wise, ascending, descending, functional, etc [15]... In this context, the A/D-UF profile has been among those investigated in little research [6]. This type of profiling seems to help maintain the filling of the intravascular volume status in patients during HD, and further adjusts weighing intervals in keeping with the filling volume of the vessels. In addition, it prevents many complications for the duration of HD induced by hypotension, as well as insufficient interdialytic weight gain (IDWG), and failure to reach dry weight at the end of each session [16]. One other method for preventing and improving hypotension, utilized along with the UF profile, is sodium profiling [17]. Thus, the combination of the UF and sodium profiles makes HD patients’ BP more stable, so there is less decrease in the BP level [18]. Sodium profiling is not applied or manually increased or decreased at the bedside in most centers, which raises some problems, such as thirst and IDWG [13]. Upon adjusting sodium profile, the HD procedure starts with hypernatremic solution at the beginning of each session, and the amount of sodium solution is diminished during the treatment, so the excess sodium transferred for the hypernatremic period is removed from the patient’s blood. Besides, it prevents more hypotension during HD by maintaining intravascular volume [19]. Recent studies have advocated the combination of the UF and sodium profiles to reduce numerous complications arising in HD [19, 20]. For example, the linear UF-sodium profiling had improved BP in the HD patients in one study by Borzou et al. (2015) [14]. The present study aims to investigate the combined effect of A/D-UF and sodium profiling on HD patients with hypotension, considering the lack of research in using these methods to improve blood pressure in Iran.

## Methods

### Trial design

**Fig. 1:**
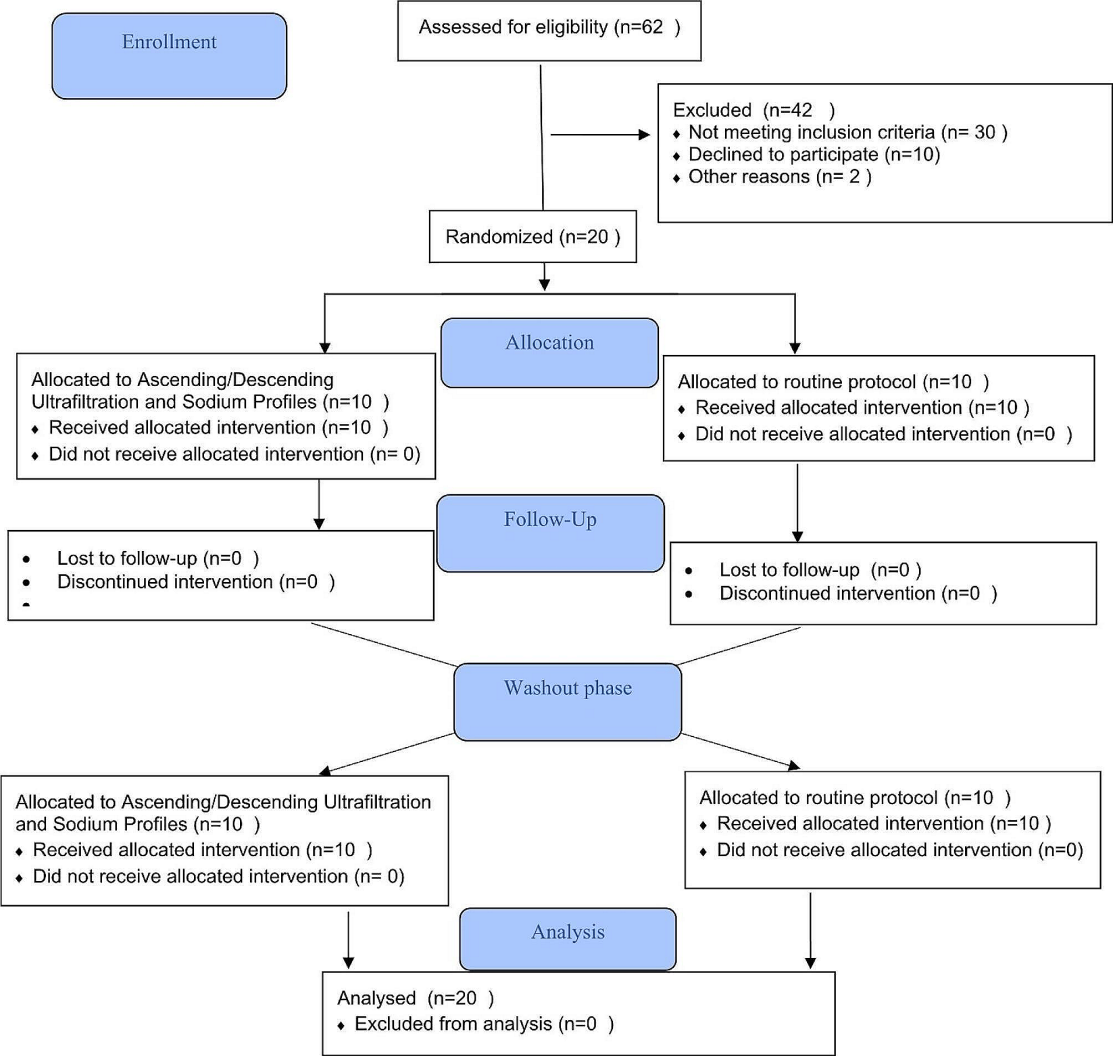
CONSORT Flow Chart of participants

Using a crossover design, this clinical trial was conducted on the HD patients referred to the Hemodialysis Center of 9-Day Hospital, Torbat-e Heydarieh, Iran, between December 2022 and June 2023 (Fig. 1).

### Participants

The inclusion criteria were being at the age range of 18–75, suffering from end-stage renal disease (ESRD), experiencing hypotension during HD in more than 20% of the sessions within one month before the study commencement, undergoing HD for over six months, having no shortness of breath and pulmonary edema, and receiving dialysis solution with sodium bicarbonate three times a week. If the patients had taken antihypertensive medications on the day of HD, the results of those sessions were excluded from the data analysis process. Likewise, BP following blood transfusion and the use of other blood volume expanders were not included.

Hypotension during HD was accordingly defined in this study as a condition wherein the systolic BP (SBP) in the patients dropped by more than 30% below 100 mmHg, compared with that before this procedure, or the diastolic BP (DBP) was below 60 mmHg. 

**Intervention**.

Two HD protocols were implemented via a crossover design for both study groups. In this respect, 16 HD sessions were considered for each patient in each protocol. The protocols were (a) an intervention protocol in which the liquid sodium in the dialysis solution was linear and the UF profiling was A/D, and (b) a routine protocol or HD, wherein both liquid sodium and UF in the dialysis solution remained constant. Besides, four wash-out HD sessions were utilized between these two protocols.

**Fig. 2:**
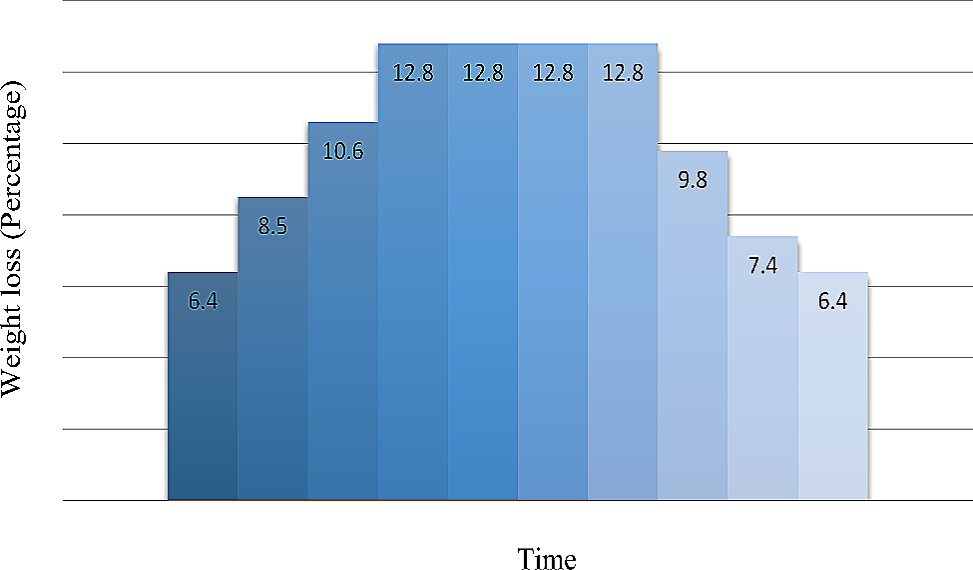
Ultrafiltration ascending-descending profile

The HD duration in the A/D-UF profiling was about four hours. In this HD protocol, UF was divided into three phases, viz., ascending, intermediate, and venular. At the ascending phase, 25.5% of the patient’s total weight was taken with a low UF rate. There was then an aggressive phase taking 51.2% of the patient’s weight at the intermediate phase, which was the maximum UF rate. At the descending phase, 23.6% of the total weight of the patient was further taken, and the UF rate was low. The UF rate refers to the rate at which a fluid or solution is filtered through a membrane using ultrafiltration. These phases were performed during ten steps using the B BRAUN Dialog plus Dialysis Machine, in profile one (Fig. 2) (Table 1).

**Table 1:** Steps of ultrafiltration ascending/descending

Phases	Steps	Time (Minutes)	Weight loss (Percentage)	UF rate (ml/h)
Initial phase (Ascending)	1	24	6.4	640
	2	24	8.5	850
	3	24	10.6	1060
Middle Phase	4	24	12.8	1280
	5	24	12.8	1280
	6	24	12.8	1280
	7	24	12.8	1280
Final phase (Descending)	8	24	9.8	950
	9	24	7.4	296
	10	24	6.4	256

**Fig. 3:**
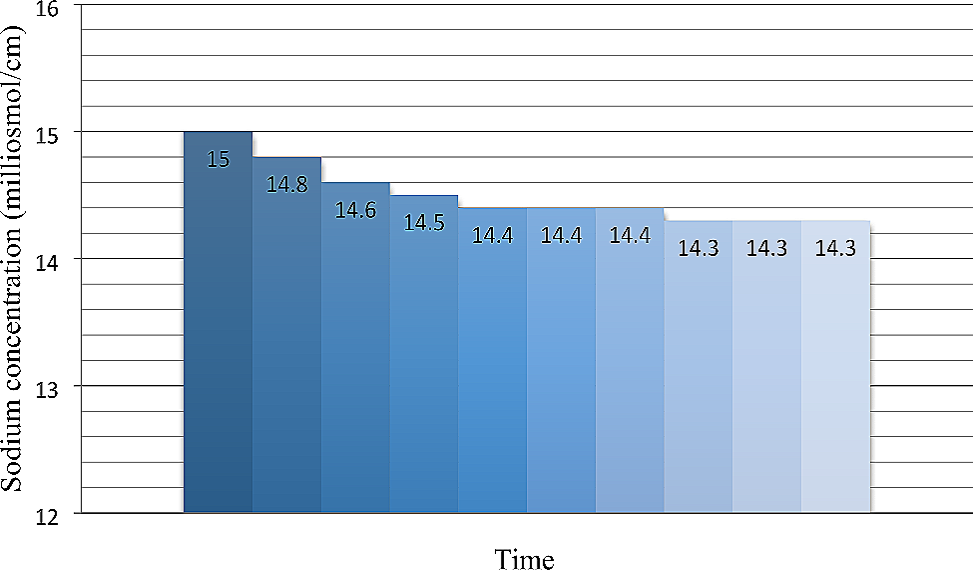
Linear profile of sodium

For the linear sodium profiling, the sodium concentration of the dialysis solution was 150 mmol/L at the onset of HD, which diminished linearly and reached 138 mmol/L at the end of the session. This HD protocol was further divided into three phases, within ten steps, each one lasting 24 min (Fig. 3) (Table 2).

**Table 2:** Steps of sodium linear profile

Phases	Steps	Time (Minutes)	Sodium concentration of dialysis solution (Ms/cm)
First Phase	1	24	15.0
	2	24	14.8
	3	24	14.6
Second Phase	4	24	14.5
	5	24	14.4
	6	24	14.4
	7	24	14.4
Third Phase	8	24	14.3
	9	24	14.3
	10	24	14.3

During the routine HD protocol, UF remained constant. The same volume of the dialysis was further removed in every hour of this procedure. In this protocol, sodium in the dialysis fluid was constant (140 mmol/L) all through a four-hour session.

Patients’ BP was then checked and recorded at six intervals, namely, before HD, one, two, three, and four hours after it, and following this procedure, within each session.

### Outcomes

A two-part questionnaire was also administered to collect the data, that is, the first part was associated with the demographic characteristics, viz., age, gender, education, income, place of residence, first HD date, and vascular access, and the second part was the BP measurement checklist.

To ensure the accuracy of the study, the B BRAUN Dialog plus Dialysis Machine (Germany) was used for all samples. The dialysis solution was sodium bicarbonate buffer and its temperature was set at 37^o^C for all patients, the blood flow rate was between 200 and 350 ml/min, and the dialysis fluid flow rate was set at 500 ml/min.

To validate the credibility of the study findings, the patients’ BP was initially measured by the researcher and other colleagues, recruiting 10 samples, and the correlation coefficient of the measured BP levels was then determined. This value was equal to 0.93 and 0.89 as reported by the researcher and the first and second collaborators, respectively. To measure BP, the same standard mercury sphygmomanometer was used for all samples.

### Sample size and randomization

Our intention is to enroll 20 patients in the study. The statistical calculations used to determine the required sample size were specifically tailored for cross-over studies, with a significance level (α) of 0.05 and a power (β) of 0.80 [21].

After extracting the baseline characteristics of the HD patients with reference to their records, those with hypotension in at least more than 20% of the sessions (that is, greater than three sessions) during the last one month, meeting the inclusion criteria in this study, were selected. The patients were then randomized into two groups of 10, benefiting the web-based randomization service (http://randomozation.com) to generate the random allocation sequence.

### Statistical methods

The data were then analyzed using the IBM SPSS Statistics 20. To describe and categorize the data, descriptive statistics, viz., frequency distribution, mean, and standard deviation (SD) were exploited. Additionally, the repeated measures analysis of variance (RM-ANOVA), paired t-test, and independent samples t-test were employed to test the research hypothesis. The normality of the quantitative variables was further established via the Kolmogorov-Smirnov (K-S) test. Of note, the 95% confidence interval (CI) and the 0.05 significance level were applied in all tests.

## Results

In total, 20 patients, including 12 men (60%) and 8 women (40%), with the mean age of 58.00 ± 14.54 on HD for an average of 54 months, were recruited in this study. The vascular access in the majority of the patients (70%) was through a fistula. The modality of most patients was High Flux Hemodialysis (85%). In this study, each patient completed 16 HD sessions, and 320 sessions were totally analyzed (Table 3).

**Table 3:** Demographic characteristics of the studied patients

Variable		N (%)			Mean				
Age (Year)			20 (100)			58.00±14.54			
Dialysis duration (Month)			20 (100)			54.55±34.77			
Modality of HD	High Flux Hemodialysis (HF-HD)			17 (85)			-		
	Low Flux Hemodialysis (LF-HD)			3 (15)			-		
Gender	Man			12 (60)			-		
	Female			8 (40)			-		
Vascular access type	Fistula			14 (70)			-		
	Vascular graft			4 (20)			-		
	Catheter			2 (10)			-		
The cause of Renal Disease	Hypertension			5 (25.0)			-		
	Diabetes			8 (40.0)			-		
	Hereditary			1 (5.0)			-		
	Polycystic kidney			4 (10.0)			-		
	Other			2 (10.0)			-		
BMI (Kg/m^2^)					24.4± 4.0				

The study results demonstrated no statistically significant difference in the mean SBP in the group with the A/D-UF profiling at the intervals before HD, one, two, three, and four hours after it, and following this procedure, in each session (*p* > 0.05). This suggested that the patients did not experience some changes in the systolic and diastolic BP levels during HD. In contrast, there was a statistically significant difference in the mean SBP in the routine HD group before this procedure, one, two, three, and four hours after it, and following HD, in each session (*p* < 0.05). In addition, SBP in the patients in the routine HD group fell by 20 mmHg until the end of this procedure (Table 2). The mean DBP of the group receiving the A/D-UF profile at the times before HD, one, two, three, and four hours after it, and following this procedure in each session showed no statistically significant difference (*p* > 0.05). This denoted that the patients did not face changes in DBP during HD. However, there was a statistically significant difference in the mean DBP in the routine HD group before HD, one, two, three, and four hours after it, and following this procedure, in each session (*p* < 0.05), and the patients had been subjected to hypotension during HD (Table 4).

**Fig. 4:**
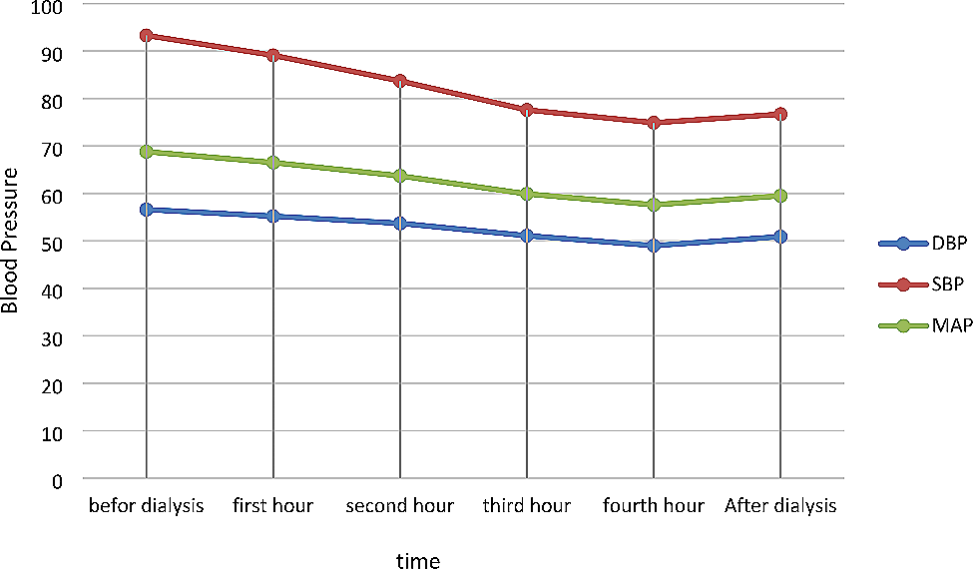
Blood pressure in Routine Profile

**Fig. 5:**
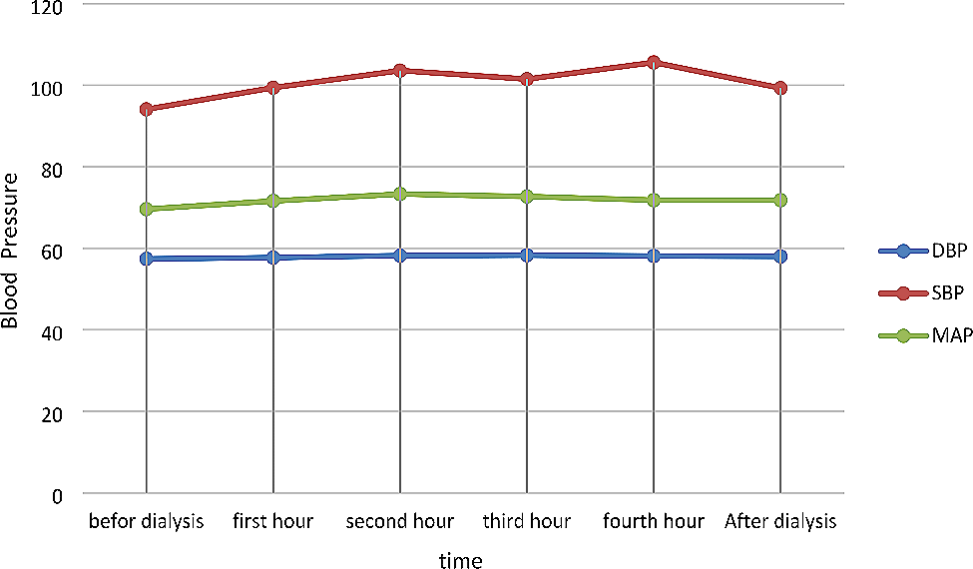
Blood pressure in ascending-descending profile

A statistically significant difference was further observed in the mean arterial pressure (MAP) in the A/D-UF profiling group before dialysis, one, two, three, and four hours after it, and after HD, in each session (*p* < 0.05). The MAP of the routine HD group before dialysis, one, two, three, and four hours after it, and following this procedure, in each session, also indicated a statistically significant difference (*p* < 0.05) (Table 4) (Fig. 4) (Fig. 5).

**Table 4:** The average blood pressure of the studied patients in the two groups of ascending-descending and routine profiles

Variable	Before dialysis	first hour	second hour	third hour	Fourth hour	After dialysis	P
Ascending Descending Profile							
SBP (95% CI)	94.09 (91.7, 96.1)	99.4 (96.8, 102.0)	103.6 (100.1, 107.1)	101.5 (97.7, 105.5)	105.6 (95.9, 119.7)	99.3 (96.2, 102.4)	0.065
DBP (95% CI)	57.4 (55.9, 58.6)	57.7 (56.1, 59.1)	58.2 (56.7, 59.3)	58.3 (56.9, 59.4)	58.1 (56.8, 59.4)	58.0 (56.7, 59.1)	0.117
MAP (95% CI)	69.6 (67.9, 71.3)	71.6 (69.7, 73.5)	73.3 (71.3, 75.3)	72.7 (70.6, 74.8)	71.8 (69.8, 73.8)	71.8 (69.9, 73.6)	*P*<0.001
Routine							
SBP (95% CI)	93.3 (90.7, 95.5)	89.1 (86.6, 91.4)	83.7 (81.0, 86.1)	77.6 (74.4, 80.3)	74.9 (71.9, 77.7)	76.7 (74.1, 79.3)	*P*<0.001
DBP (95% CI)	56.6 (55.4, 57.9)	55.2 (53.9, 56.5)	53.7 (52.1, 55.2)	51.1 (49.5, 52.8)	49.0 (47.3, 50.8)	50.9 (48.9, 52.7)	*P*<0.001
MAP (95% CI)	68.8 (67.3, 70.3)	66.5 (64.9, 67.9)	63.7 (61.8, 65.3)	59.9 (57.8, 61.8)	57.6 (55.6, 59.7)	59.5 (57.5, 61.3)	*P*<0.001

Table 5 presents a comparative analysis detailing the various aspects of treatment administered to two distinct groups: one following an ascending-descending profile approach and the other adhering to a routine or standard treatment protocol. This table aims to elucidate the differences in treatment characteristics between these methodologies. Our cross-over clinical trial demonstrated a statistically significant reduction in symptomatic IDH episodes from 55 to 15% with the application of the A/D-UF profile (*p* < 0.05) (Table 5).

**Table 5:** Characteristics of treatment in two groups: ascending-descending profile and routine

Variable		Ascending Descending Profile No (%), Mean ± SD or Median (Min.Max.)	Routine No (%), Mean ± SD or Median (Min.Max.)	P	
Dialysis Duration (hr)		4.0 (3.0–5.0)	4.0 (3.0–5.0)	-	
Dialysate temperature (°c)		36.50 (35.0–37.20)	36.50 (35.0–37.20)	-	
Blood fow rate (ml/min)		300.0 (200.0–450.0)	300.0 (200.0–450.0)	-	
Total UF (ml/session)		3227.4 ± 1355.6	3680.6 ± 933.5	-	
Average UFR ( ml/kg/h)		10.25 (8.5–12)	11.25 (9.5–13)	-	
Dry Weight (Kg)		67.6 ± 12.9	67.6 ± 12.9	-	
Pre Dialysis Weight (Kg)		70.3 ± 13.2	70.8 ± 13.6	-	
Post Dialysis Weight (Kg)		67.6 ± 12.9	67.9 ± 12.9	-	
Sodium Levels Pre Dialysis (mEq/L)		136.1 ± 3.5	136.8 ± 3.9	Intragroup comparison in the routine group	**P < 0.001
Sodium Levels Post Dialysis (mEq/L)		140.3 ± 2.2	138.5 ± 2.2	Intragroup comparison of A/D profiles	**P < 0.001
IDWG		2.7 ± 1.3	3.1 ± 0.9	-	
Heart Rate		74.7 ± 5.5	73.2 ± 5.9	-	
Serum Albumin (g/dl)		3.9 ± 0.8	3.8 ± 0.3	-	
Number of symptomatic intradialytic hypotension		18 (15%)	66(%55)	* *P* = 0.002	
Concomitant treatment during dialysis for IDH	Midodrine	32 (27%)	34 (28%)		
	no concurrent treatment	88 (73%)	86 (72%)		

*Independent Samples T-Test ** Paired t test

## Discussion

Hypotension is among the major complications arising during HD, which has been further acknowledged as the leading cause of discomfort in the patients affected [19]. The results of the present study accordingly established that the patients’ BP in the group receiving the A/D-UF profiling did not drop during HD, but remained in the same range until the end. Nevertheless, the patients’ BP in the routine HD group gradually dropped, and this condition aggravated up until the session completed.

One of the main concerns related to the use of the UF profiling for HD has been thus the interdialytic increase in BP, which seems to be a rise in BP before this procedure. In the present study, no significant difference was further observed in the mean SBP among the patients before HD in the A/D-UF profiling group. Thus, it was concluded that the application of the A/D-UF profile did not produce a sudden increase in the interdialytic BP, and this alleviated the concerns about the growth in BP following this treatment method, which had been until that time brought up in some studies.

Upon the review of previous research into the effect of the A/D-UF profile on patients’ BP during HD, no similar study was found. Therefore, the results of other investigations in this domain were considered. As an example, Tang et al. (2016) assessed the effect of the linear sodium profiling on BP in 13 HD patients at a hospital in China, and presented that the mean SBP after HD was higher in the intervention group than the controls [22], which was consistent with the results of the present study. This type of profiling seemed to help maintain the filling of the intravascular volume status in patients during HD, and further adjusted weighing intervals in keeping with the filling volume of the vessels. In addition, it prevented many complications for the duration of HD induced by hypotension, as well as insufficient interdialytic weight gain (IDWG), and failure to reach dry weight at the end of each session [16]. Moreover, Ghafouri-Fard et al. (2010) reflected on the effect of the linear and step-wise sodium and UF profiles on hypotension and muscle cramps on 26 patients undergoing HD at a hospital in Isfahan, Iran, and showed that the use of both profiles as trouble-free and low-cost methods could stabilize the patient’s hemodynamic status during HD by adjusting the sodium concentration and the UF extraction rate [3].

As well, Borzou et al. (2015) questioned the effect of the linear sodium-UF profiling on tolerance in HD, and confirmed that hypotension was significantly lower in this method as compared with the conventional ones. Additionally, convenience was higher in the linear sodium-UF profiling [14]. Making some changes in the sodium concentration and the amount of fluid withdrawal enhancing vascular refilling in the linear sodium-UF profiling could thus fuel the patients’ tolerance in HD [23], which was in line with the results of the present study. Molaie et al. (2014) also investigated the effect of UF and sodium concentration of the dialysis solution in the prevention of BP and muscle cramps during this procedure, and indicated that the sodium and UF profiles as simple and inexpensive methods could reduce hypotension-related complications and muscle cramps [24].

In this context, Meira et al. (2010) compared two types of sodium profiles, namely, linear and stepwise, on complications during HD in 22 patients in Brazil, and showed that BP and muscle cramps in the linear and step-wise profile group were lower than those advocated in the conventional ones [25]. These findings were in harmony with the results of the present study. Coli et al. (2003) correspondingly deemed that the UF profiling could result in hemodynamic stability in patients and even prevent blood volume loss during HD as well as hypotension [26]. Moreover, Maksimov (2002) maintained that BP in HD patients could be kept at an optimal level if a combination of the sodium and UF profiles had been practiced [27].

Our study observed the trends in patients’ BP levels when subjected to the A/D-UF profiling as opposed to the routine HD protocol. The former group showed a stability in BP, which could suggest an indirect indication of the protocol’s potential in maintaining intravascular volume status. Nevertheless, it should be considered that the A/D-UF-Na group might have experienced lower IDWG and lower UF rates, which in turn may have contributed to the higher BP observed in the study. It is necessary to note that while our study did not directly measure changes in IDWG or precisely analyze the intravascular volume status, the findings related to BP stability imply a potentially beneficial profile that merits further investigation to elucidate this relationship. Future studies specifically designed to assess the impact of UF rate and IDWG on BP outcomes will provide the clarity required to affirm these findings definitively.

In light of the statistical significance in sodium concentration changes before and after dialysis in the A/D-UF group, we delve deeper into its physiological repercussions. Although the A/D-UF profile is not entirely sodium-neutral, the apparent stability it provides could be attributed in part to a vasopressin-mediated effect linked with higher plasma sodium concentration. Ettema’s research elucidates this association by demonstrating that increased plasma sodium levels can stimulate vasopressin release, enhancing vascular tone and volume status [28]. This osmotically-driven vasopressin release could be one of the underlying mechanisms contributing to the improved hemodynamic stability observed in our study with the A/D-UF protocol.

Overall, the utilization of the A/D UF and linear sodium profiles as undemanding and economical methods could stabilize the patients’ hemodynamic status during HD, and further reduce hypotension by adjusting the sodium concentration and the UF amount. Recent studies have accordingly proposed the combination of both profiles [29], but no previous research was found to investigate the effect of the A/D-UF profiling on BP among HD patients.

The limitations of this research include the unavailability of some patients (due to travel), the failure of dialysis machines, the small number of B-Brown machines in the hemodialysis center, as well as the need for further clinical investigation to assess the usefulness of the sodium-ultrafiltration profile in routine practice.

## Conclusion

As evidenced in this study, the A/D-UF profile could prevent hypotension, as well as IDWG, and failure to reach dry weight at the end of each session. From this perspective, it was suggested to use the A/D-UF profiling, compared with the conventional treatment methods, in order to prevent hypotension for the duration of HD.

## Data Availability

The datasets generated in the current study are available from the corresponding author upon reasonable request.

## Abbreviations

A/D-UF: Ascending/Descending Ultrafiltration
HD: Hemodialysis
CKD: Chronic kidney disease
BP: Blood pressure
ECF: Extracellular space
IDWG: Interdialytic weight gain
ESRD: End-Stage Renal Disease
